# Lowe syndrome identified in the offspring of an oocyte donor who was an unknown carrier of a *de novo* mutation: a case report and review of the literature

**DOI:** 10.1186/s13256-019-2263-9

**Published:** 2019-11-02

**Authors:** P. Tatsi, G. E. Papanikolaou, T. Chartomatsidou, I. Papoulidis, A. Athanasiadis, R. Najdecki, E. Timotheou

**Affiliations:** 1 Centre of Reproduction and Genetics, Assisting Nature, Thessaloniki, Greece; 2 3rd Department of Obstetrics and Gynecology, Aristotle University of Thessaloniki, Thessaloniki, Greece; 3 ATG, Access To Genome Clinical Laboratory Genetics, Thessaloniki, Greece

**Keywords:** Lowe syndrome, Dent disease, Assisted reproduction, Rare genetic disease, X-linked disease, Oocyte donation program, *De novo* mutation, IVF, Genetic screening

## Abstract

**Background:**

Oculocerebrorenal syndrome of Lowe is an X-linked disorder with very low prevalence in the general population. The *OCRL* gene encodes the protein phosphatidylinositol 4,5-bisphosphate-5-phosphatase, a lipid phosphatase, located in the trans-Golgi network. Point mutations in the *OCRL* gene cause Lowe syndrome and Dent disease, which are characterized as a multisystemic disorder. The symptoms of Lowe syndrome are expressed primarily as dysfunction of the eyes, kidneys, and the central nervous system.

**Case presentation:**

This report describes a case of a 31-year-old Georgian woman with a *de novo* pathogenic mutation causing oculocerebrorenal syndrome of Lowe, who was a volunteer in an oocyte donation program for *in vitro* fertilization purposes, and the outcome of the treatments of this particular donor’s oocyte receivers, describing the implications of the mutation for the children born as a result of the treatments. It raises important medical and ethical issues about the necessity of genetic testing of oocyte donors and the possibility of rare genetic disorders being inherited by the offspring of donors.

**Conclusion:**

This particular case indicates the legal, medical, and emotional risks of utilizing donor oocytes from phenotypically healthy women, whose genetic constitution is unknown in terms of being silent carriers of rare diseases. In addition, all the necessary actions were followed; the further examinations that are required are mentioned. The donor and the offspring should be further tested. The remaining cryopreserved embryos should be destroyed or preimplantation genetic testing should be performed before they are utilized. Finally, all the people involved, the treated couples and the donor, alongside her family, should follow genetic and psychological counselling.

## Introduction

Oculocerebrorenal syndrome of Lowe (OCRL) or Lowe syndrome is an X-linked disorder [1], first described in 1952 [2]. The syndrome is extremely rare; its estimated prevalence is 1 out of 500,000 individuals in the general population [3].

Lowe syndrome is related to a combination of: (i) ocular anomalies (cataracts, glaucoma); (ii) renal disorders; and (iii) central nervous system defects (hypotonia, hyporeflexia, intellectual disability). It is also correlated with oral manifestations: delayed eruption, crowding, and constricted palate; taurodontism of the molars; hypoplastic enamel; dental caries; and large pericoronal radiolucencies [4].

### Genetic and molecular basis of the syndrome

**Fig. 1:**
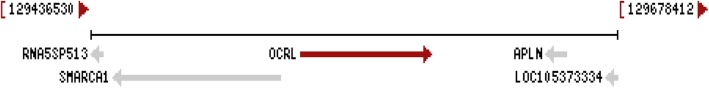
*OCRL* gene, Online Mendelian Inheritance in Man database

**Fig. 2:**
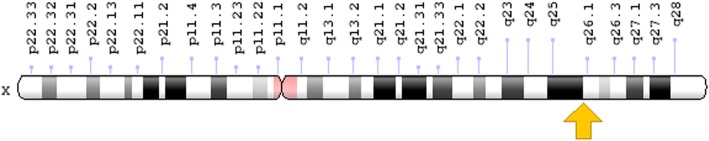
*OCRL* gene location in X chromosome (Xq26.1), Online Mendelian Inheritance in Man database

Lowe syndrome is caused by mutations in the *OCRL* gene (Figs. 1 and 2), which is located on chromosome Xq26 (#300535) [5, 6]. To date, more than 200 mutations in the *OCRL* gene (2680 bp) have been described. The genetic basis could be nonsense mutations, splice site mutations, missense mutations, insertions, and deletions [7]. Normally, the *OCRL* gene encodes the protein phosphatidylinositol 4,5-bisphosphate-5-phosphatase (OCRL-1), which is a lipid phosphatase, localized in the trans-Golgi network [8, 9]. The importance of the protein relies on the fact that it controls the cellular levels of a critical metabolite, phosphatidylinositol 4,5-bisphosphate (PIP_2_). Thus, the mutated gene, which results in the deficiency of the enzyme, causes the protean manifestations of the syndrome. In fact, cells affected by Lowe syndrome, and consequently tissues and organs, are characterized by disturbed metabolism because PIP_2_ is crucial in intracellular signaling, protein trafficking, and polymerization of actin cytoskeleton [3, 9, 10]. It is suggested that the accumulation of the metabolite, caused by the enzyme insufficiency, along with a mutual disequilibrium of the phosphoinositides lead to the clinical picture of the affected infants.

Mutations in the *OCRL* gene also result in Dent disease type 2 (#300555). Dent disease type 2 is characterized by a broad phenotypic spectrum, depending on the *OCRL* mutations that are responsible, implying that this particular disease may be a mild variant/phenotype of Lowe syndrome [11].

### Diagnostic criteria – differential diagnosis

Point mutations in the *OCRL* gene cause the multisystemic disorder Lowe syndrome, affecting the eyes, kidneys, and the central nervous system [2, 9]. All patients suffering from Lowe syndrome show dense bilateral cataracts present at birth. Half of the cases develop glaucoma, which can be detected within the first year of life or, in some cases, later in life [3]. The renal dysfunction is expressed as Fanconi syndrome, which can be detected during the first months of life and shows a wide range of severity among the population. The symptoms include proteinuria, proximal renal tubular acidosis, bicarbonaturia, phosphaturia, hypercalciuria, aminoaciduria, and hypokalemia [12]. The above symptoms may lead during the second decade of life to chronic renal failure [3].

The first picture of the syndrome is apparent at birth, as serious hypotonia and hyporeflexia are observed. A general delay in neuropsychomotor development is also observed. Approximately 10% of the patients with Lowe syndrome show mild to severe intellectual disability [3]. Lowe syndrome is associated with maladaptive behavior, including auto-aggressive and hetero-aggressive behaviors, anger, and obsessive-compulsive behavior [13]. Approximately 50% of the adult patients show seizures and 9% of the patients present febrile convulsions [14, 15].

Among the reported symptoms of Dent disease type 2, patients usually display low molecular weight proteinuria and hypercalciuria and in some cases nephrocalcinosis. Additional symptoms may include increased lactate dehydrogenase, increased creatine kinase, short stature, and umbilical hernia [11]. At least six different mutations of the *OCRL* gene have been described and it is estimated that 59.7% of families showing a Dent disease phenotype have mutations of the *OCRL* gene [11].

## Case presentation

### Oocyte donation program

The Georgian donor at the age of 31 years visited Assisting Nature *in vitro* fertilization (IVF) clinic and expressed her desire to donate her oocytes to infertile couples. According to Greek law, thorough medical screening of all female donors is required: (i) blood group, Rhesus type, hemoglobin (Hb)-electrophoresis; (ii) genetic tests for cystic fibrosis, α thalassemia, β thalassemia, δβ thalassemia and Fragile X syndrome, and full karyotype testing; and (iii) testing for infectious diseases is also mandatory, that is, human immunodeficiency virus (HIV), Venereal Disease Research Laboratory (VDRL) antigen, hepatitis B virus (HBV), and hepatitis C virus (HCV). We additionally perform: (iv) hormonal evaluation, that is, anti-Müllerian hormone (AMH) level, estradiol, progesterone, follicle-stimulating hormone (FSH)/luteinizing hormone (LH) levels; (v) full blood account, biochemical tests, and urine test; and, finally, (vi) Minnesota Multiphasic Personality Inventory® 2.0 psychological test. The donor underwent all the necessary medical examinations. She was found negative in all necessary laboratory examinations; her genetic testing was normal and after checking her family medical history, no inherited genetic disorders were found. Her fertility was also proven by the fact that she is the mother of a healthy young girl. As a result, since all the necessary procedures were followed, she was accepted as a donor in the donation program of our clinic and after thorough consultation she signed informed consent to donate her oocytes anonymously as stated in Greek law.

This particular donor participated in the clinic’s egg donation program by donating her oocytes four times during a period of 18 months beginning February 2016. An antagonist protocol with 225–300 IU of recombinant FSH (rec-FSH) or human menopausal gonadotropin (hMG) was used in all four ovarian stimulations and recombinant human chorionic gonadotropin (rec-hCG) or gonadotropin-releasing hormone (GnRH) agonist was administered to trigger ovulation; the oocytes were collected 36 hours later and were donated to five couples seeking fertility treatment with egg donation.

### Acceptors treatment and offspring follow-up

The first couple received 11 oocytes of which 10 were mature and 7 blastocysts were formed. On the day of the embryo transfer, two blastocysts were transferred and five were vitrified. Nine days post embryo transfer, a human chorionic gonadotropin (hCG) test was positive, a twin pregnancy was later confirmed by ultrasound, and finally two healthy male infants were delivered.

The second couple received nine oocytes that were all fertilized and cultured until day 5 blastocyst stage. At day 5, two blastocysts were transferred and five were vitrified. The hCG result test was positive, but the pregnancy was terminated at 22 weeks due to craniofacial malformation. One year later, a frozen embryo transfer (FRET) cycle was performed in which two blastocysts were thawed; the blastocysts survived and were transferred which resulted in a clinical pregnancy and delivery of a healthy boy.

The third couple received six oocytes, from which four blastocysts were formed. Two of them were transferred and the surplus two were vitrified. The result was positive and after 8 months two babies, of both sexes, were delivered and reported to be healthy. Further communication with the third couple was not feasible and the offspring were not tested for the mutation.

The fourth couple received four oocytes, which after fertilization formed four blastocysts. All of them were vitrified for 2 months, since the cycles of the donor and the acceptor were unsynchronized. The subsequent FRET cycle resulted in a twin pregnancy. This pregnancy resulted in a preterm birth of two boys at 24th week. Unfortunately, the first infant died during the first week after the delivery. The second preterm baby presented clinical manifestations, such as cataract and renal abnormalities, which led the neonatologists into a genetic investigation of the neonate. Six months later the tests revealed the genetic origin of the disease indicating Lowe syndrome. At this point, the couple informed us about the case and the chance that the biological mother-donor might be the carrier of a mutation that causes Lowe syndrome.

The fifth couple who received oocytes from this donor had already undergone embryo transfer by the time we were informed about the possible Lowe syndrome from the previous couple. They also achieved a twin pregnancy, leading to delivery of twins: a healthy baby girl and a boy that suffers from some of the optical symptoms of Lowe syndrome. The parents informed us that the boy had already been operated on for cataracts. We are expecting the genetic analysis of the potentially affected boy.

In conclusion, 41 metaphase II (MII) oocytes were used from this particular donor and 31 utilizable blastocysts were formed. Nine offspring were delivered by the treated couples resulting in a live birth rate of 75% since 12 blastocysts were embryo transferred. Of all the children delivered seven were male and two were female. The two female children were unaffected by the mutation; out of the nine male offspring, two were diagnosed with Lowe syndrome and one died, its diagnosis remains unknown, and, finally, the rest of them were healthy and unaffected by the disease.

### Genetic counselling of the donor

**Fig. 3:**
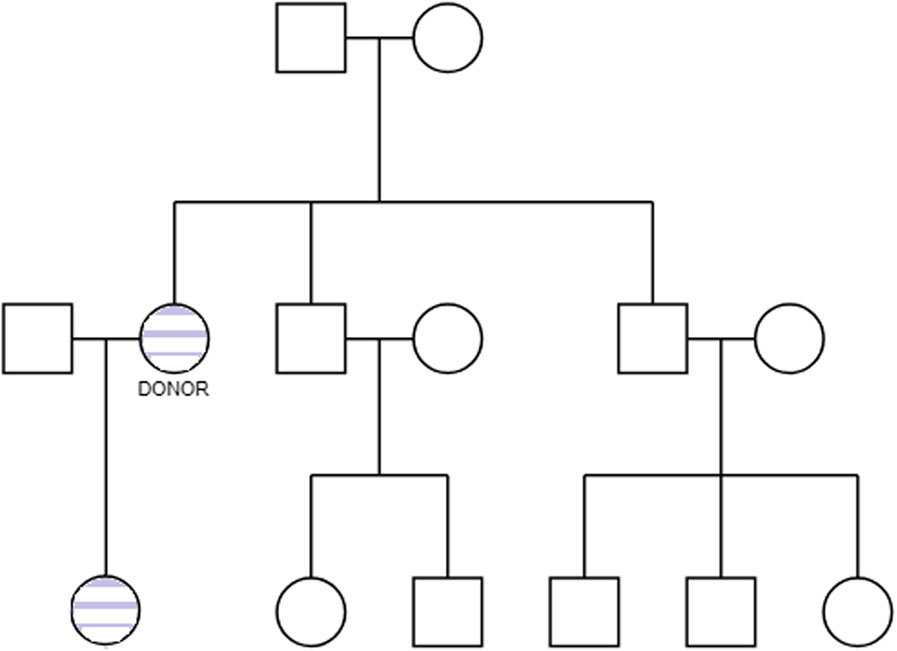
A hereditary pedigree showing the affected members of the family. Donor (carrier of a *de novo OCRL* mutation) and her daughter (also carrier). No other family member (even her two brothers) is affected by Lowe syndrome or carries the mutation causing Lowe syndrome or Dent disease

The donor was immediately informed, and after discussion about protecting her anonymity, she agreed to undergo additional genetic analysis. Supplementary counseling was offered: first, with regard to her female offspring’s future family planning; and, second, regarding her wish for a possible future pregnancy. The extra genetic tests that the donor underwent proved that a *de novo* mutation in the *OCRL* gene of the X chromosome exists, despite the absence of any previous family medical history of Lowe syndrome, and it is responsible for Lowe syndrome and Dent disease (Fig. 3 Hereditary tree).

### Genetic analysis

A genetic laboratory conducted the analysis from genomic deoxyribonucleic acid (DNA) isolated from a peripheral blood sample. The analysis included sequencing of the whole coding region and all of the splicing regions of exons and introns (±8 bp) of the gene *OCRL* (NM_000276.3, chr. X). The amplification of the examined regions was completed by oligonucleotide-based target capture (QXT, Agilent Technologies) followed by next generation sequencing (MiSeq, Illumina). Alignment and variant calling were performed using Burrows-Wheeler Aligner (BWA, MiSeq reporter) and Genome Analysis Toolkit (GATK). Sanger sequencing was performed in order to confirm any detected variant. Results from this analysis were evaluated according to international standards [16] and included all mutations with known clinical significance, mutations non-reported in the literature, and mutations with unknown clinical significance.

The genetic analysis proved c.940-11G>A p.? mutation in *OCRL* gene in heterozygous condition. According to international literature, pathogenic mutations to *OCRL* gene cause Lowe syndrome (#309000) and Dent disease (#300555) in an X-linked recessive inheritance pattern. The c.940-11G>A p.? mutation is reported in patients with Lowe syndrome and affects splicing procedure resulting in the premature termination codon (p.Lys313_Val314insAsnSer*) that alters the structure and function of the produced protein.

By the time we received the genetic results of the donor the Greek National Authority of Assisted Reproduction was informed for further actions to be implemented. In addition, we communicated with all the couples involved with this donor to ask them to inform us about the health status of their children, whether they are affected by Lowe syndrome or not and to provide genetic counseling (Table 1).

**Table 1:** Summary of the cases treated with the affected donor’s oocytes

Cases	MII oocytes	Blasts available	Fresh ET	Result	FRET ET	Live birth	Gender	Affected	Outcome
Couple 1	10	7	2D5	Pregnant	–	2	Male-male	Unaffected	Healthy-healthy
Couple 2	9	7	2D5	Terminated	2D5	1	Male	Unaffected	Healthy
Couple 3	6	4	2D5	Pregnant	–	2	Male-female	Unaffected	Healthy-healthy
Couple 4	4	4	–	–	2D5	2	Male-male	Unknown-affected	1 boy died, 1 alive
Couple 5	12	9	2D5	Pregnant	–	2	Male-female	female unaffected, male affected	Boy operated cataracts

*2D5* 2 embryos day 5, *ET* embryo transfer, *FRET* frozen embryo transfer, *MII* metaphase II

## Discussion

The above case signifies the legal and emotional risks of utilizing donor oocytes from phenotypically healthy women, whose genetic constitution is unknown in terms of being silent carriers of rare diseases. First, such genetic risks cannot be avoided, and second, although the couples who receive the donor eggs are extensively consulted, it is difficult for them to realize or even accept taking such a risk [17, 18].

Procreation with donor oocytes is expected to be as safe as the natural conception of fertile couples. Therefore, although the possibility of having a child with an inherited disorder is still present, oocyte acceptor couples underestimate such a risk. On the other hand, currently, extensive genetic testing of gamete donors is not recommended for economic or ethical reasons, unless there is a family history or other specific reason [18]. In our specific donor, the mutation is very rare (1 out of 500,000) and, moreover, none of her male family members were affected, indicating the *de novo* origin of the mutation.

Since Lowe syndrome is an X-linked disease, it is usually inherited by sons from the mother. Asymptomatic female individuals may be carriers of the mutated gene and carry 25% probability of having a child (boy) with the syndrome. Carriers of Lowe syndrome can be reliably (94%) diagnosed by an ophthalmological evaluation [19], as they display specific lens opacities on slit-lamp examination [20]. However, such a diagnostic tool is usually used only in cases of women with a family history of the syndrome because they are at greater risk of having an affected child. The family of this particular donor had never shown any relevant symptoms and, as a consequence, no such examination took place.

Cases of *de novo* mutations have been reported in 30% of male individuals with Lowe syndrome. In this case, the mother is not a carrier and the possibility of having another affected child is equal to that of the general population, as proved to happen in this specific case. Similarly, somatic/germline mosaicism may be identified and should be considered and mentioned in genetic counseling (4.5% of the patients) [21]. In cases of women with a germline mosaicism, no molecular, enzymatic, or slit-lamp testing can be used for identification of the situation and the women are perfectly healthy. In cases of female carriers, measurement of the activity of the enzyme is not usually accurate because of the random X-chromosome inactivation [22]. In any case, mothers of affected boys or women with a family history of Lowe syndrome should undergo prenatal testing.

It is obvious that if all the necessary examinations and tests of a candidate donor are negative and the donor has a normal status, no family record of any syndrome exists, and is considered healthy, then she is thus suitable for an oocyte donation program. Syndromes with extremely rare emergence, such as Lowe syndrome, can practically only be diagnosed at a preimplantation or prenatal level in families with a known history of the disease. Otherwise, in cases in which prenatal testing is required, it can be performed with DNA analysis and ultrasound examination. In the latter case, ultrasonography aims to detect the early presence of fetal cataracts. The fetal lenses can be identified from 14 weeks and high attention should be given to the orbital region during the ultrasound [23]. Alternatively, fetal cells obtained either by chorionic villus sampling or by amniocentesis can be used for DNA analysis for the *OCRL1* gene.

In the unfortunate coincidence that the prenatal diagnosis of the syndrome has not been done and an ailing child is born, then the treatment of Lowe syndrome is mainly symptomatic, aiming to improve the clinical condition and the evolution of the disease. One of the major goals of the treatment is to postpone the terminal renal disease, which is the most frequent reason for death [3] and limits the lifespan of the patients to a maximum of 40 years. Management of the ocular dysfunctions includes surgical cataract removal (as already happened in one of the cases), use of glasses, and frequent testing for early glaucoma diagnosis. In addition, rehabilitation therapy and seizure drugs are required to treat the complications of the syndrome, while other drugs such as neuroleptics, stimulants, anti-depressives, and so on can be used, but their efficiency is limited. Renal dysfunctions may be treated with different supplements and vitamin D is needed to treat rickets. In any case, the right treatment should be decided according to the clinical status of the patient. Only a minority of patients are recorded to have had a successful renal transplantation [3].

The importance of psychological counseling in fertility care has been acknowledged for many years because infertility treatment is stressful and may result in multiple losses that provoke feelings of sadness, grief, depression, anxiety, and isolation. IVF treatment with donor eggs is highly successful but social, legal, and ethical issues arise. Screening and educating patients about the possible implications of gamete donation have become central recommendations for pretreatment counseling in guidelines issued in the USA, UK, Australia, New Zealand, and Germany among other countries. Internationally, considerable variation remains in recipient counseling, ranging from legislated, recommended, to minimal [24, 25].

## Conclusions

The aforementioned realistic case indicates that, in the near future, carrier screening of oocyte or sperm donors might become fundamental. Ropers in 2012 stated that 1–2% of all couples are carriers of genetic diseases and in this case a 25% risk of having an affected child exists [26]. In the extreme scenario that a full genetic screening (WGS) should be performed, oocyte donors may be excluded from egg donation programs for possible risks that may never emerge. All donors are plausible carriers of such conditions so targeted carrier testing might be preferable to extended screening.

Thorough genetic consultation is of paramount importance for: (i) donors, to scrutinize their hereditary history and provide useful information about family members and therefore early identification of possible genetic diseases; and (ii) acceptors of genetic material in order to realize the risks that such procedures render. The current case of a rare disease – Lowe syndrome – is such an excellent paradigm that carrier screening of donors in the future, although still controversial for ethical reasons due to genetic selectivity, might gain a lot of popularity.

## Data Availability

Available on request.

## Abbreviations

AMH: Anti-Müllerian hormone
FRET: Frozen embryo transfer
FSH: Follicle-stimulating hormone
GATK: Genome Analysis Toolkit
GnRH: Gonadotropin-releasing hormone
Hb: Hemoglobin
HBV: Hepatitis B virus
hCG: Human chorionic gonadotropin
HCV: Hepatitis C virus
HIV: Human immunodeficiency virus
hMG: Human menopausal gonadotropin
IVF: *In vitro* fertilization
LH: Luteinizing hormone
MII: Metaphase II
*OCRL*: *OCRL* gene
OCRL: Oculocerebrorenal syndrome of Lowe
PIP_2_: Phosphatidylinositol 4,5-bisphosphate
protein OCRL-1: Phosphatidylinositol 4,5-bisphosphate-5-phosphatase
rec-FSH: Recombinant follicle-stimulating hormone
rec-hCG: Recombinant human chorionic gonadotropin
VDRL: Venereal Disease Research Laboratory
WGS: Full genetic screening
